# Understanding D-Ribose and Mitochondrial Function

**DOI:** 10.7575/aiac.abcmed.v.6n.1p.1

**Published:** 2018

**Authors:** Diane E. Mahoney, John B. Hiebert, Amanda Thimmesch, John T. Pierce, James L. Vacek, Richard L. Clancy, Andrew J. Sauer, Janet D. Pierce

**Affiliations:** 1 University of Kansas Medical Center, School of Nursing, Kansas, US; 2 University of Kansas Medical Center, Kansas, US; 3 Center for Advanced Heart Failure and Heart Transplantation, Kansas, US

**Keywords:** Adenosine Triphosphate, Bioenergetics, D-ribose, Mitochondria

## Abstract

Mitochondria are important organelles referred to as cellular powerhouses for their unique properties of cellular energy production. With many pathologic conditions and aging, mitochondrial function declines, and there is a reduction in the production of adenosine triphosphate. The energy carrying molecule generated by cellular respiration and by pentose phosphate pathway, an alternative pathway of glucose metabolism. D-ribose is a naturally occurring monosaccharide found in the cells and particularly in the mitochondria is essential in energy production. Without sufficient energy, cells cannot maintain integrity and function. Supplemental D-ribose has been shown to improve cellular processes when there is mitochondrial dysfunction. When individuals take supplemental D-ribose, it can bypass part of the pentose pathway to produce D-ribose-5-phosphate for the production of energy. In this article, we review how energy is produced by cellular respiration, the pentose pathway, and the use of supplemental D-ribose.

## INTRODUCTION

Mitochondria are among the most important organelles in cells. They function as the cell powerhouse because 99% of adenosine triphosphate (ATP) is produced within mitochondria, and ATP is the main energy source for intracellular metabolic pathways (1–3). Mitochondrial dysfunction can produce extreme fatigue and other symptoms that are common complaints among patients, especially those individuals with heart failure. The reduction in mitochondrial function at the cellular level is often associated with loss of both the electrical and chemical transmembrane potential, the alteration of the electron transport chain function, and diminished transport of mitochondrial metabolites needed for cellular function (2,4,5).

The mitochondria are considered to have developed from an ancient synergy in which a nucleated cell was engulfed by an aerobic prokaryote. In this endosymbiotic relationship, the host eukaryote gradually transformed into a mitochondrion using oxygen to produce energy (6). Mitochondria also contain their own deoxyribonucleic acid (DNA) and transcriptional and translational mechanisms. Certain diseases are now being associated with mitochondrial DNA (mtDNA) defects that contribute to underlying energetic factors (7,8). D-ribose is a naturally occurring monosaccharide within the pentose pathway that assists with ATP production. It is a 5-carbon chain (also called aldopentose) and is a key component of DNA, ribonucleic acid (RNA), acetyl coenzyme A, and ATP (9). Cells produce D-ribose through the pentose phosphate pathway (PPP) that is essential for ATP production. In many diseases or conditions, ATP synthesis is reduced, thus supplementation with D-ribose may provide a solution to impaired cellular bioenergetics (10). In this article, we review how energy is produced by cellular respiration, the pentose pathway, and the use of supplemental D-ribose.

### Mitochondria

Mitochondria are highly dynamic double membrane-bound organelles (cellular components) found in the cytoplasm of most eukaryotic cells, those cells that contain a nucleus (11–14). The primary function of mitochondria is to provide chemical energy required for cellular biosynthesis through their vibrant abilities to convert energy from nutrient molecules and store this energy in phosphate bonds within a molecule known as ATP (15–17).

Synthesis of ATP (also known as bioenergetics) within mitochondria is essential for producing the energy needed for normal cellular processes. In addition to energy generation, mitochondria have a role in other mechanisms including cellular metabolism, calcium signaling, and cell death (18,19). Mitochondria also contain some DNA material, although the majority of genomic data reside within cell nuclei (17). The quantity of mitochondria needed is dependent upon energy demands. For example, cells requiring more energy, such as those found in skeletal muscles, possess greater quantities of mitochondria (20).

Mitochondria are oval shaped structures that vary in size and distribution to meet cellular requirements (21). Each mitochondrion has a double membrane composed of proteins and phospholipids (22,23). The double membranes of the mitochondrion form four distinct components within the organelle: (1) a smooth contour outer membrane; (2) an intermembrane space; (3) an inner membrane; and (4) a matrix. The outer membrane creates a physical border between the mitochondrion and the rest of the cell (24). This membrane is formed by a single phospholipid bilayer containing proteins called porins that enable permeability for free passage of other proteins such as ATP, ions, and nutrient molecules (16). The intermembrane space is the region between the outer and inner membranes. The matrix is enclosed within the intermembrane and contains a host of proteins and enzymes involved in ATP synthesis and genetic material. The inner membrane is a highly complex structure consisting of numerous infoldings that are organized into sophisticated layers known as cristae. In contrast to the outer membrane, the inner membrane does not contain porins and is highly impermeable to most molecules. As a result, ions and molecules require specialized transporters for selective membrane passage (25,26).

### Cellular Respiration

**Figure 1:**
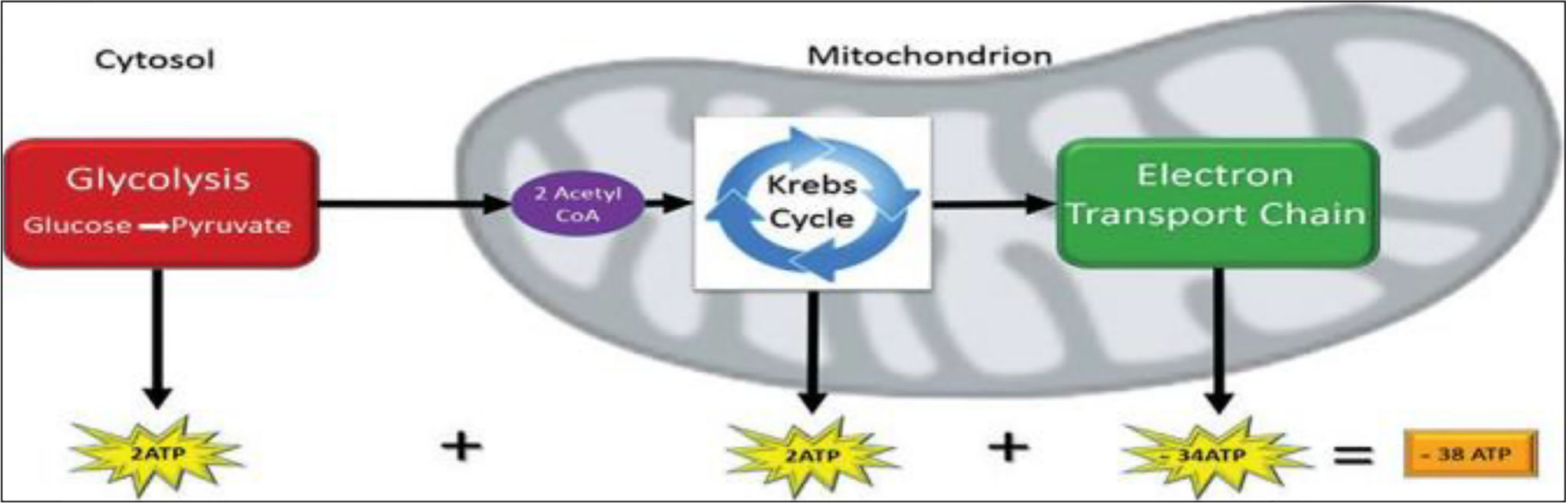
The stages of cellular respiration include glycolysis, pyruvate oxidation, Krebs cycle, and electron transport chain.

Cellular respiration is a series of biochemical reactions within mitochondria that results in ATP production (27). Adenosine triphosphate is generated through a highly organized system embedded within the inner membrane. Cellular respiration involves three processes: (1) glycolysis; (2) the citric acid cycle, also known as the Krebs cycle; and (3) the electron transport chain, also referred to as oxidative phosphorylation (Figure 1) (28).

Glycolysis is the anaerobic pathway within the cell cytoplasm in which glucose, a six carbon sugar, is converted into two molecules made of three carbons called pyruvate. From this pathway, one glucose molecule yields two ATP molecules (29). Following glycolysis, pyruvate enters the mitochondrion, and enzymatic systems in the mitochondrial matrix convert pyruvate into two carbon molecules called acetyl-CoA(28).

Acetyl-CoA then enters the citric acid cycle and undergoes a series of biochemical reactions with enzymes that yield carbon dioxide and electron carrier molecules known as nicotinamide adenine dinucleotide (NADH) and flavinadenine dinucleotide (FADH_2_). There are two additional ATP molecules produced for each glucose molecule undergoing glycolysis. The majority of ATP is generated from the last phase of cellular respiration, an aerobic pathway known as the electron transport chain (30).

The electron transport chain consists of a group of complex proteins, referred to as protein complexes I to IV that are housed within the mitochondrial inner membrane (31). Hydrogen electrons from NADH and FADH_2_ molecules pass through the transport chain from one complex to another creating a proton gradient across the membrane. Energy transfer from electrons is used to pump protons across the membrane space throughout the entire cristae surface. Once the concentration gradient of electrons becomes higher in the membrane space, protons migrate to the area of lower concentration in the mitochondrial matrix via an enzyme known as ATP synthase (32). This enzyme catalyzes the production of ATP by the phosphorylation of adenosine diphosphate (ADP). The electron transport chain pathway generates approximately 34 additional ATP molecules. Thus, cellular respiration yields approximately 38 ATP molecules from one glucose molecule (Figure 1) (33).

### D-ribose

**Figure 2:**
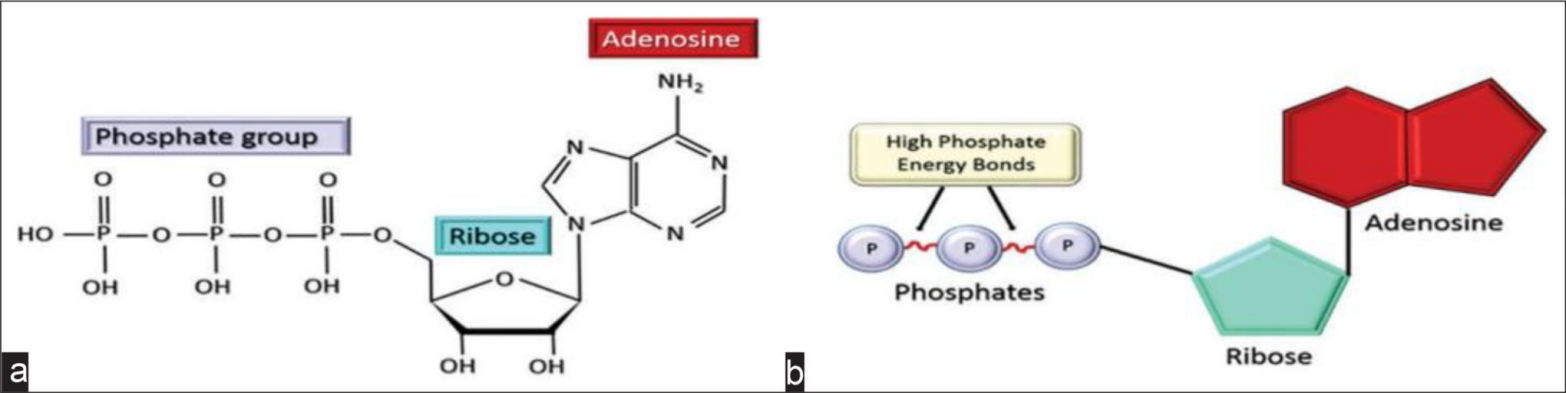
(a) Chemical structure of adenosine triphosphate (ATP); (b) The ATP molecule is composed of an adenosine ring and a ribose sugar with three phosphate groups. From the high energy bonds among the phosphate group, ATP is produced.

D-ribose is an energy producing substrate of the ATP molecule and is often called the “molecular currency” because of its role in intracellular energy transfer. Adenosine triphosphate consists of phosphate, ribose, and adenosine groups that are connected through two high-energy phosphoanhydride bonds within the molecule. (Figure 2) (34).

**Figure 3:**
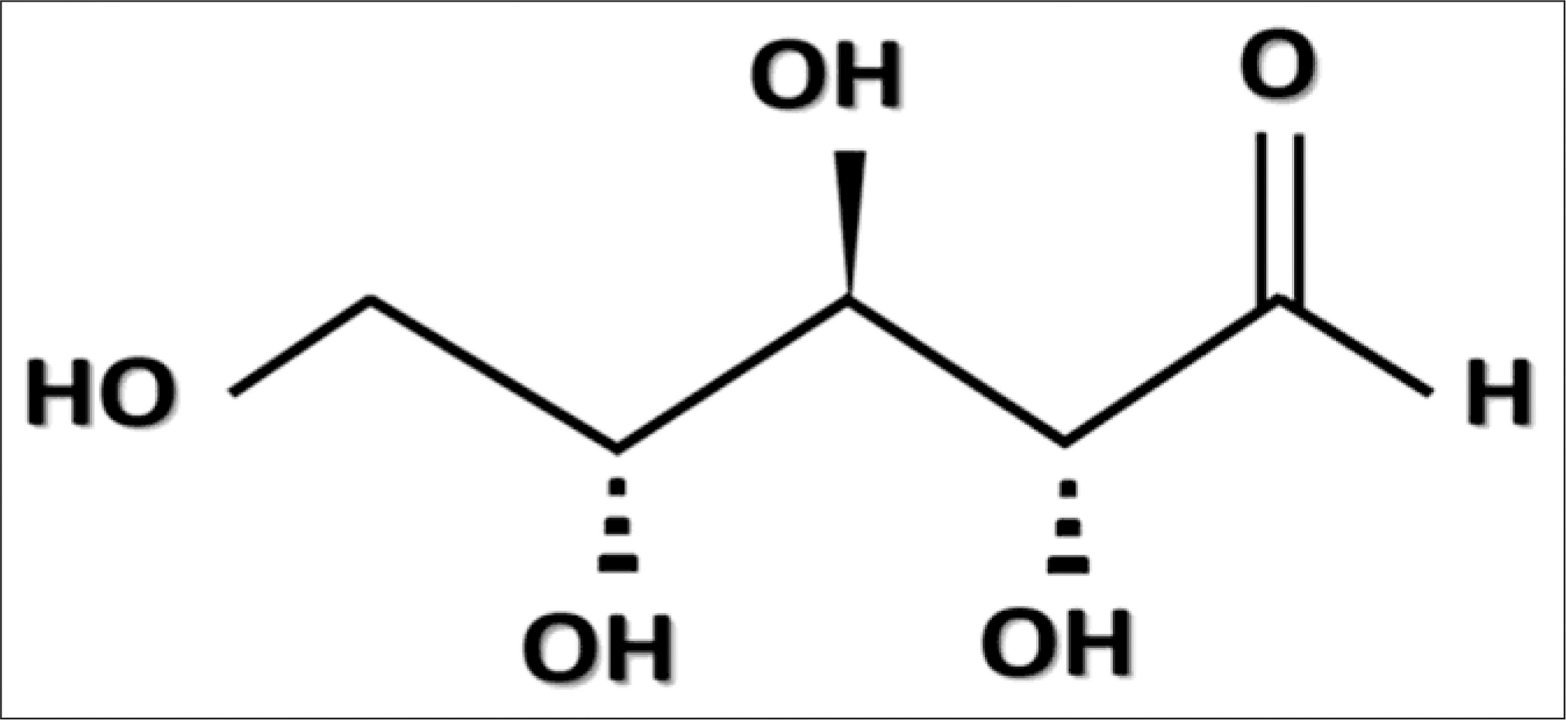
An image of the chemical structure of D-Ribose which is a pentose monosaccharide (simple sugar).

As a pentose sugar, D-ribose has five carbons in its ring structure; the chemical structure (Figure 3), and the molecular weight is 150.13 g/mol (35).

**Figure 4:**
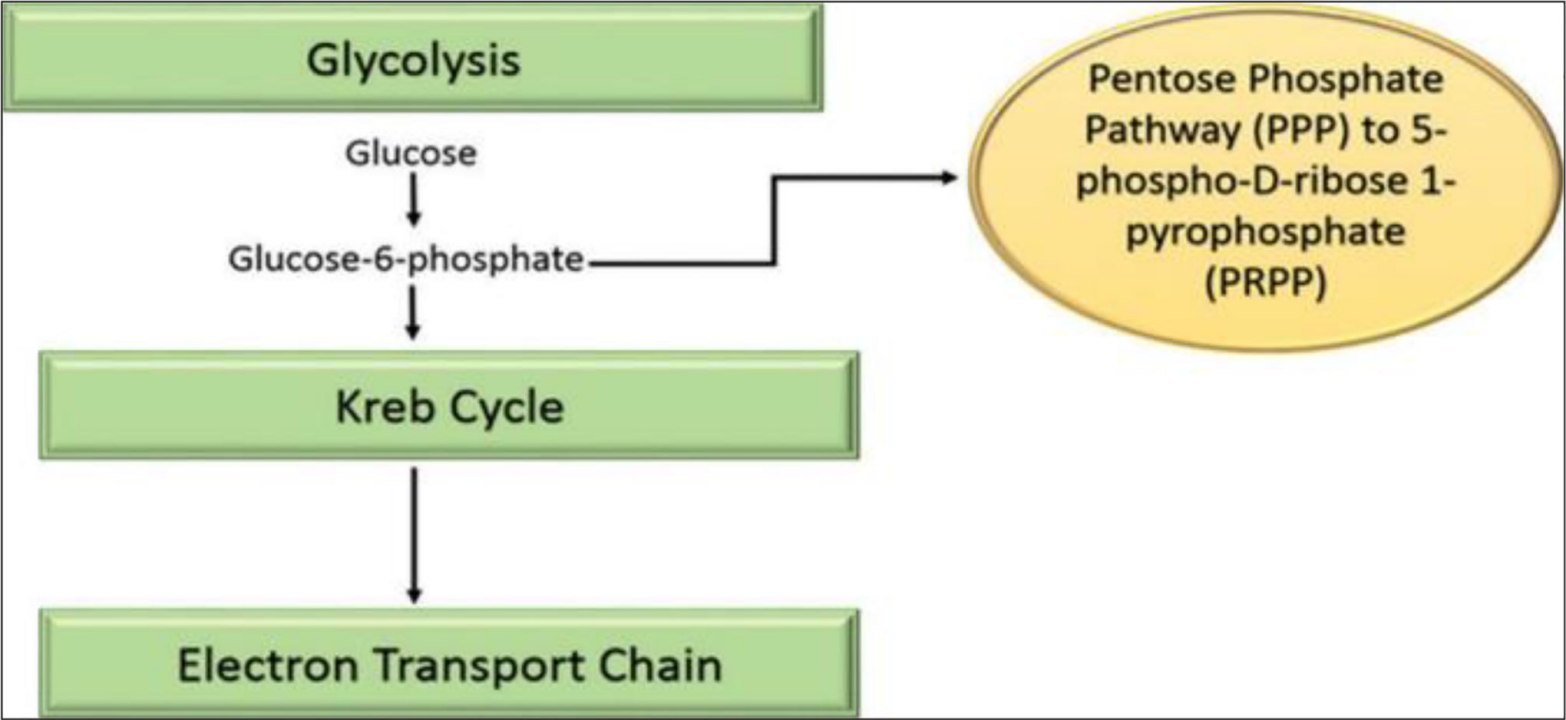
The relationship of the pentose phosphate pathway to stages of cellular respiration.

The ATP molecule is able to store and transport chemical energy within cells and is essential for synthesis of nucleus acids such as DNA and RNA. Ribose is a naturally occurring five-carbon sugar produced in the body through the PPP, a metabolic pathway parallel to glycolysis that generates nicotinamide adenine dinucleotide phosphate (NADPH), pentoses, and ribose 5-phosphate. The PPP is a slow process that requires an enzyme called glucose-6-phosphate dehydrogenase (G-6-PDH) that is often in short supply within the cells (Figure 4). This enzyme can have limited expression in the myocardial cells with cardiac disease leading to significant delay in the production of ribose (36).

There are two main pathways — the de novo and salvage—for the synthesis of nucleotides. Using the 5-phosphoribo-syl-1-pyrophosphate (PRPP), the de novo pathway enzymes build purine and pyrimidine nucleotides from the beginning of the process with ribose. This pathway is much slower than the salvage pathway in which preformed ribose allows the cells to quickly and efficiently recycle the ATP end products. The mitochondria use the ATP metabolites to form new ATP for energy production. Consequently, ribose is crucial for both the de novo and salvage pathways (37–39).

### Supplemental D-ribose

In certain pathologic conditions such as heart failure, cellular energy deficiency exists in myocardial mitochondria. The reduction in ATP production is directly correlated with the decreased supply of D-ribose in the mitochondria. This may be related to the limited expression of the G-6-PDH enzyme in myocardium that can significantly interrupt the production of ribose. Several studies have shown that augmenting D-ribose following myocardial ischemia improves mitochondrial function by increasing myocardial ATP production (40).

**Figure 5:**
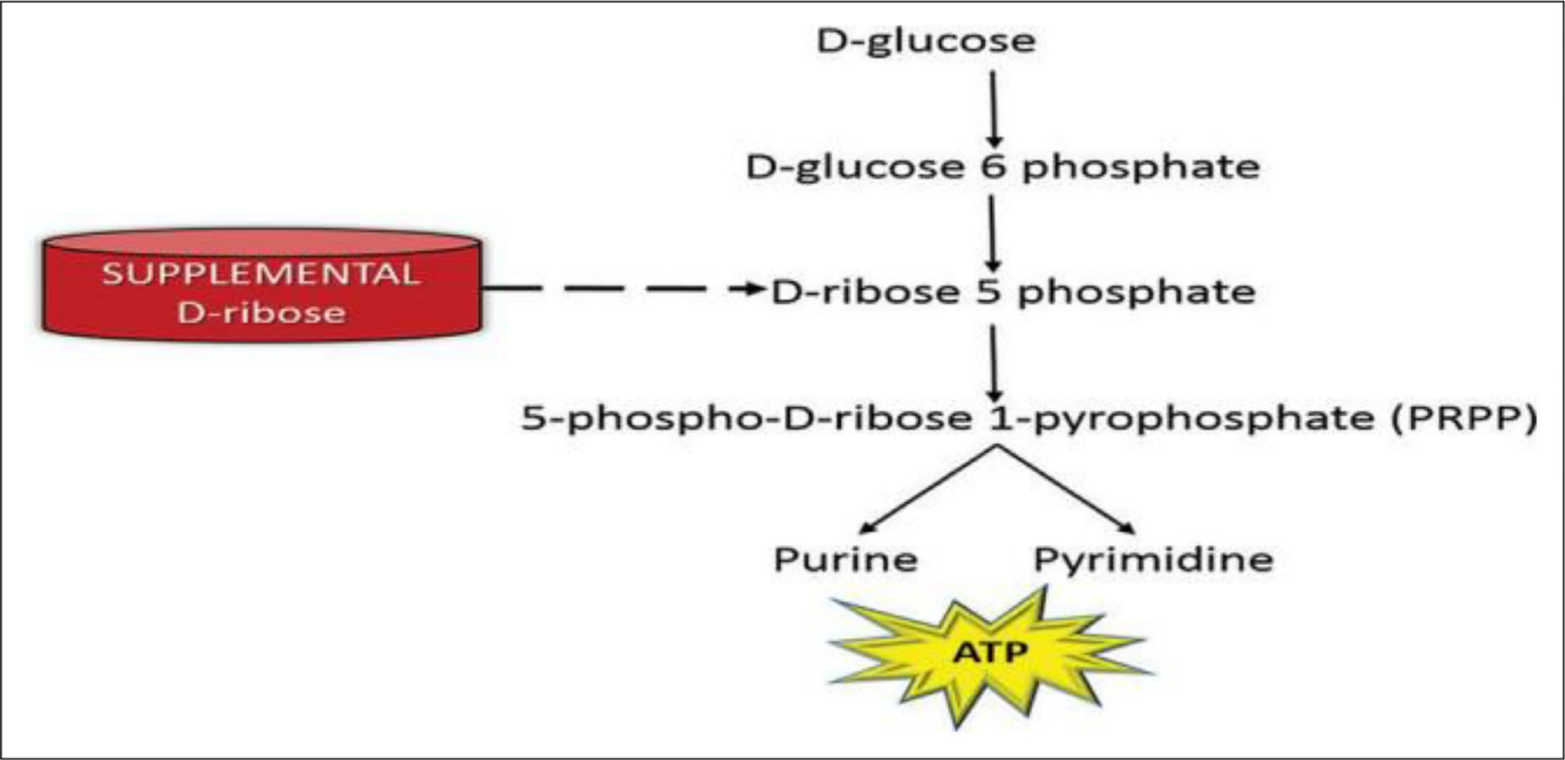
Supplemental D-ribose bypasses the upper part of the pentose pathway and is an alternative source for 5-phospho-D-ribose 1-pyrophosphate (PRPP).

Administering supplemental D-ribose circumvents the enzymatic step to assist with replenishing ATP levels in cells. In purine metabolism, D-glucose is transformed to both D-ribose-6-phosphate and to D-ribose-5-phosphate and then altered to 5-phospho-D-ribose 1-pyrophsophate (PRPP) for the synthesis of purine and pyrimidine (Figure 5). In other words, supplemental D-ribose bypasses the rate-controlling PPP (slower pathway) and provides an alternate source of PRPP for ATP production.

D-ribose has been used both orally and intravenously in patients for many different pathologic conditions such as chronic fatigue syndrome (41), fibromyalgia (42), and myocardial dysfunction (40). It is often used to improve athletic performance and reduce symptoms of cramping, pain, and stiffness following exercise (41). Under different pathologic conditions, ATP, ADP, and adenosine monophosphate are degraded and not available for energy production. Supplemental D-ribose has been shown to enhance recovery of ATP levels and reduce cellular injury in humans and animals (9,43). A study by Pliml et al. found that patients with severe coronary artery disease who consumed D-ribose for 3 days had improved myocardial tolerance to ischemia. They hypothesized that supplemental D-ribose increased ATP metabolism and assisted with restoring cardiac energy metabolism (44). Another group of investigators found that daily oral D-ribose significantly improved left a trial function in congestive heart failure patients. They demonstrated that supplemental D-ribose not only improved diastolic performance but also improved the patient’s physical activity function and quality of life.

Supplemental D-ribose can be purchased in a dry powder form, and the recommended dose ranges from 5 to 15 grams per day and not by body mass units (42). The powder is mixed in a non-carbonated drink and has a sweet taste. It is readily metabolized if consumed within 30 minutes after being mixed in fluid. The side effects are minimal, but patients have reported mild diarrhea, slight nausea, and stomach discomfort that was reduced by consuming the drink with food (45–47). There had been some concerns about the safety of ribose therapy related to the inhibitory effects of ribose on cell proliferation in vitro. However, Pliml et al. investigated possible side effects of ribose on human lymphocytes. They found no significant inhibition of human lymphocyte proliferation in vitro in mitogen-stimulated cells and no evidence that ribose therapy was harmful to human cells (48).

## CONCLUSION

Mitochondria regulate a multitude of metabolic and signaling pathways, but their primary function is the production of ATP. When mitochondrial function is compromised, there can be a reduced efficiency of cellular respiration and thus a loss of ATP production. D-ribose is an ATP substrate naturally occurring within cells. When nucleotides are reduced, supplemental D-ribose has been shown to be useful in enhancing the recovery of these energy molecules. Thus, D-ribose supplementation may help to return adenine nucleotides to the cell and thereby serve as a potential therapeutic option for various pathophysiologic conditions.
